# Head and Neck Ultrasound Utilization Rates: 2012 to 2019

**DOI:** 10.1002/oto2.97

**Published:** 2023-11-22

**Authors:** Courtney B. Shires, John D. Boughter, Aaron Smith, Merry E Sebelik

**Affiliations:** ^1^ West Cancer Center Germantown Tennessee USA; ^2^ Department of Neuroanatomy University of Tennessee Memphis Tennessee USA; ^3^ Advanced ENT and Allergy Louisville Kentucky USA; ^4^ Department of Otolaryngology–Head and Neck Surgery Emory University Atlanta Georgia USA

**Keywords:** head and neck, ultrasound, utilization

## Abstract

**Objective:**

We measured utilization of clinician‐performed head and neck ultrasound among otolaryngologists, endocrinologists, and general surgeons, using Medicare Provider Utilization and Payment Data.

**Study Design:**

Retrospective analysis of Medicare billing database.

**Setting:**

University.

**Methods:**

For each year, the files were filtered to include 4 provider types: Diagnostic Radiology (DR), Endocrinology (ENDO), General Surgery (GS), and Otolaryngology (OTO). Billable procedures are listed by Healthcare Common Procedure Coding System code and a filter was applied to include 76536 Ultrasound, soft tissues of the head and neck.

**Results:**

In 2019, OTOs submitted charges for 2.1% of all head and neck diagnostic ultrasounds (76536) performed on Medicare beneficiaries. For each year 2012 to 2019, DRs submitted the most charges, followed by ENDOs, and then OTO and GS. Charges for all groups increased in a proportional manner across the 8‐year period. 14.5% of OTOs submitted more than 100 charges apiece during 2019, that is, “super users.” The percentage of super users within each specialty increased from 2012 to 2019. Overall, the data support an ever‐increasing use of head and neck ultrasound (HNUS) among all provider types.

**Conclusion:**

Even with increased use among OTOs, this specialty only accounted for a small percentage of head & neck diagnostic ultrasounds performed on Medicare beneficiaries in 2019. Changes in volume of nonradiology point‐of‐care HNUS was not associated with changes in DR volume. A greater proportion of OTOs than DRs are “super users” among the ultrasound users within their specialty, performing more than 100 exams/year.

**Level of Evidence:**

V.

Clinician‐performed ultrasound of the head and neck is a valuable tool to gain timely access to diagnostic imaging, facilitate image‐guided procedures such as biopsy and therapeutic injections, and to enhance the value of the patient‐physician visit. Increasingly, training opportunities are available to the nonradiologist, in the form of head and neck‐focused ultrasound courses offered by professional specialty societies,^1^ with positive impact on patient care.^2^

Several subspecialties perform head and neck ultrasounds on a routine basis including general surgery, otolaryngologists, radiologists, and endocrinologists. However, the utilization of this powerful tool by each of these subspecialties is largely unknown. This study seeks to measure the magnitude of utilization of clinician‐performed ultrasound among otolaryngologists, general surgeons, endocrinologists, and diagnostic radiologists.

## Methods

**Figure 1:**
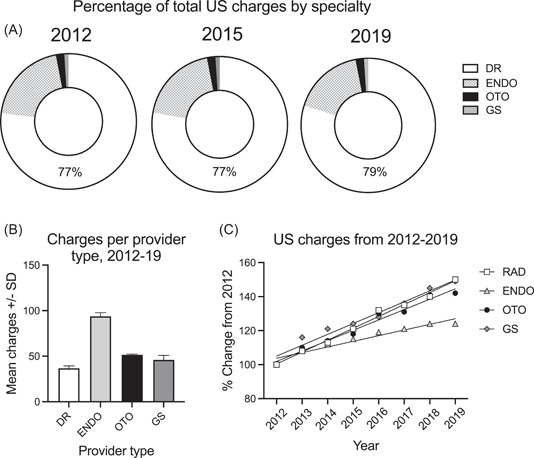
Head and neck ultrasound charges by specialty from 2012 to 2019. (A) Percentages according to specialty. (B) Average number of charges per provider within each specialty. (C) Rate of change of US charges plotted for specialties from 2012 to 2019.

We queried the Medicare Provider Utilization and Payment Data: Physician and Other Supplier Public Use File (PUF) (Figure 1) available through the Centers for Medicare & Medicaid Services (CMS.gov).^3^ Billable procedures are listed by Healthcare Common Procedure Coding System (HCPCS) code; we quantified incidence of the HCPCS billing code 76536—Ultrasound, soft tissues of the head and neck (eg, thyroid, parathyroid, parotid), real‐time with image documentation. As 76942—Ultrasonic guidance for needle placement (eg, biopsy, aspiration, injection, localization device), imaging supervision and interpretation, and 10022—Fine needle aspiration, with imaging guidance, are not specific to the head and neck, these 2 HCPCS codes were not included.

For each of the years available in the dataset (2012‐2019), the files were filtered to include 4 provider types: Diagnostic Radiology (DR), Endocrinology (ENDO), General Surgery (GS), and Otolaryngology (OTO).

Descriptive statistics were calculated for all 8 years. Comparisons among provider type and across year were also made via 1‐ or 2‐way analysis of variance (ANOVA). Additionally, percentage values across years were fitted with simple linear regressions to determine if the slopes were nonzero, indicating significant change with time. Statistical tests were performed using GraphPad Prism 9.4.0 (GraphPad Software LLC). As this is an analysis of a public database, institutional review board exemption was obtained from Emory University.

## Results

### HNUS Charges by Specialty

Number of submitted charges, according to specialty, for code 76536 over the time period of 2012 to 2019 are listed in Table 1. Radiologists (DR) accounted for the vast majority of charges (77%‐79%) in any 1 year, followed by the non‐Radiologist provider types: ENDO (17%‐20%), OTO (2%), and GS (1%). These proportions did not vary significantly from year to year (Figure 1A); A 2‐way ANOVA (provider type × year) indicated a significant effect of provider type (*F*_[3,21]_ = 20,556, *P* < .0001), but not year (*F*_[7,21]_ = 0.25, *P* = .97). The absolute numbers of charges per specialty was related to the overall number of providers of each type submitting these charges (DR > ENDO > OTO > GS), which are listed in Table 2. However, the average number of US charges per provider type (Figure 1B) per year was greatest for ENDO, at 93.86 ± 3.97 (mean ± SD), followed by OTO (51.56 ± 0.85), GS (46.09 ± 2.62), and DR (36.87 ± 2.62). For this measure, there was a significant effect of provider type (1‐way ANOVA; *F*_[3,31]_ = 439.9, *P* < .0001); ENDO had almost twice as many charges per provider than the other 3 specialties.

**Table 1:** Submitted Charges by Year, by Specialty, for Code 76536 (Diagnostic Ultrasound of Head and Neck)

Specialty	2012	2013	2014	2015	2016	2017	2018	2019
DR	416,712	450,402	471,873	505,568	551,556	564,237	584,237	624,318
ENDO	108,024	117,068	121,082	124,502	128,044	131,038	133,709	134,054
OTO	11,464	12,585	13,020	13,490	14,859	15,011	16,181	16,324
GS	7488	8661	9049	9298	9559	10,174	10,867	11,186
Total	543,688	588,716	615,024	652,858	704,018	720,498	744,994	785,882

**Table 2:** Number of Providers of Each Specialty Using HNUS in Active Practice

Specialty	2012	2013	2014	2015	2016	2017	2018	2019
DR	12,682	13,176	13,346	13,659	14,698	14,832	14,912	15,314
ENDO	1241	1280	1320	1344	1350	1361	1375	1345
OTO	224	249	249	266	282	295	313	311
GS	191	206	216	206	201	195	222	216
Total	14,338	14,911	15,131	15,475	16,531	16,683	16,822	17,186

As mentioned above, it is evident from the data in Table 1 that the number of US charges within each specialty increased from year‐to‐year across the 8‐year period that was examined. Indeed, charges increased significantly (*Fs* > 85.45, *ps* < 0.0001) for each specialty from 2012 to 2019 (Figure 1C). This increase occurred at an equivalent rate among specialties except for ENDOs, whose increase in use was more gradual (*F*_[3,24]_ = 16.4, *P* < .0001). Collectively, these data confirm the trend for ever‐growing use of head and neck ultrasound across all disciplines.

### Super Users

**Figure 2:**
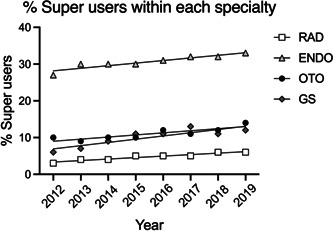
Percentage of head and neck ultrasound super users within each specialty. Percentages increased significantly within each group over the 8‐year period (*Fs* > 19.20; *ps* < 0.0047).

We next examined the number of “Super users” of US, that is, those physicians submitting ≥100 charges per year. In terms of absolute numbers, in any given year there were more super users among DR, followed by ENDO, OTO, and GS (Table 3). For example, in 2019, there were 958 super users among DR, followed by 440 for ENDO, 45 for OTO, and 26 for GS. However, when number of super users was expressed as a percentage among total providers using US (within each specialty, ie, Table 2), ENDO had the highest values ranging from 27% to 33% across the 8‐year period examined (Figure 2). The percentage of super users for OTO ranged from 9% to 14%, for GS 6% to 13%, and for DR 3 to 6. These percentages increased slightly, but significantly (*Fs* > 19.20, *ps* < 0.0047), across the period from 2012 to 2019.

**Table 3:** Number of Super Users for Each Specialty Using HNUS

Specialty	2012	2013	2014	2015	2016	2017	2018	2019
DR	430	484	546	628	730	743	838	958
ENDO	340	385	392	403	421	438	441	440
OTO	22	23	26	27	35	31	36	45
GS	11	15	19	22	25	26	24	26
Total	803	907	983	1080	1209	1238	1339	1469

## Discussion

Ultrasound examination has been likened to the “stethoscope of the fingers” and carries great value in its immediacy. The combination of portability and easy cleaning allows for ease in use. Point‐of‐care ultrasound allows same visit service and management decisions.^4^ Its use has expanded to include ultrasound examination of the thyroid gland, vocal cord motion, parotid gland, submandibular gland, lymph nodes, head and neck primary biopsy, arterial evaluation, trachea‐esophageal puncture (TEP) placement, abscess drainage, and submucosal cleft evaluation.^5^

For Otolaryngologists, the increased use of HNUS across recent years is noteworthy. For example, in 2012, 9.82% of OTOs in the PUF were classified as super users, submitting more than 100 charges apiece. In 2019, this percentage had jumped to 14.5%, as compared to the much smaller proportion of radiologists (4.5%) who did so. Among surgeons in 2019, OTOs performed more diagnostic HNUS than GSs (16324 vs 11186), and the percentage of OTOs performing US compared to their specialty peers was 3.5 times higher than GSs.

The interest in and use of ultrasound has increased across the board. From 2012 to 2019, the number of ultrasound charges submitted to Medicare/Medicaid has increased, on average, by 45% among all 4 provider types examined. Stark differences exist between the subspecialties in terms of billing procedure. Radiologists (~70%) vastly bill under a facility, while general surgeons (~20%) to a lesser degree, and endocrinologists (~8%) and otolaryngologists (~5%) much less.

In the current analysis, we show that endocrinologists averaged a higher number of ultrasounds performed than the other provider types. We believe this is due to these specialists spending more time in clinic allowing for point‐of‐care ultrasound. This belief is supported by the higher percentage (27%‐33%, from 2012 to 19) of endocrinologists that are superusers compared to other provider types (3%‐12%). Point‐of‐care ultrasound attains a higher billing rate and reimbursement compared to traditional radiologist‐performed ultrasound due to the fact that the overwhelming majority of radiology ultrasonography is performed at a facility. Our study illustrates that, once adjusting for facility versus nonfacility charging, the gap between the subspecialties lessened. Historically, the nonfacility reimbursement is roughly 3 to 4 times more, and this trend continued within this study.

As in other clinical specialties, otolaryngology has been adopting the concept of point‐of‐care ultrasound in the new millennium, while encountering barriers of time, training, confidence, and expense.^6^ There have been concerns that increasing utilization of clinician‐performed ultrasound will threaten radiology case volume and reimbursement.^7^ However, nonradiology point‐of‐care HNUS appeared to have no impact on radiology volume over the years 2012 to 2019. This is the first study that measures relative utilization between traditional radiology‐performed HNUS and that performed by point‐of‐care otolaryngologists, general surgeons, and endocrinologists.

## Conclusions

Even with increased use among OTOs, this specialty only accounted for a small percentage of head & neck diagnostic ultrasounds performed on Medicare beneficiaries in 2019. Changes in volume of nonradiology point‐of‐care HNUS was not associated with changes in DR volume. A greater proportion of OTOs than DRs are “super users” among the ultrasound users within their specialty, performing more than 100 exams/year.

## Author contributions

**Courtney B. Shires**, project concept, creating project proposal, data collection, writing manuscript, editing manuscript; **John D. Boughter, Jr.**, project concept, creating project proposal, data collection, writing manuscript, editing manuscript; **Aaron Smith**, project concept, creating project proposal, data collection, writing manuscript, editing manuscript; **Merry Sebelik**, project concept, creating project proposal, data collection, writing manuscript, editing manuscript.

## Disclosures

### Competing interests

None.

### Funding source

None.
